# Changes in living arrangements and mortality among older people in China

**DOI:** 10.1016/j.ssmph.2016.11.009

**Published:** 2016-11-30

**Authors:** Zhixin Feng, Jane Falkingham, Xiaoting Liu, Athina Vlachantoni

**Affiliations:** a Centre for Research on Ageing, School of Social Sciences, Faculty of Social, Human and Mathematical Sciences, University of Southampton, UK; b ESRC Centre for Population Change and Centre for Research on Ageing, School of Social Sciences, Faculty of Social, Human and Mathematical Sciences, University of Southampton, UK; c Department of Social Security & Risk Management, School of Public Affairs, Zhejiang University, China; d Centre for Research on Ageing and ESRC Centre for Population Change, School of Social Sciences, Faculty of Social, Human and Mathematical Sciences, University of Southampton, UK

**Keywords:** Changes in living arrangements, Elderly people, China, Mortality, Cox-proportional model

## Abstract

Living arrangements in later life are dynamic, with changes associated with life events such as widowhood or moves into an institution. Previous research has found particular changes in living arrangements to be associated with an elevated risk of mortality. However, research in this area within the context of China is limited, despite China being home to the world's largest population of older people. This study investigates the impact of changes in living arrangements on older persons’ survival using the Chinese Longitudinal Healthy Longevity Survey from 2002 to 2011. The original sample was 16,064 in 2002, and this study includes 6191 individuals who survived in 2005 and had complete information of track record in later waves. Changes in living arrangements are examined between 2002 and 2005. Cox-proportional hazards models are then used to investigate the association between the dynamics of living arrangements and respondents’ survival status from 2005 to 2011 . Results show that men and women who lived in an institution in both 2002 and 2005, or who moved into an institution from living with family faced a greater risk of dying compared to those continuing to live with family. By contrast, continuing to live with family or alone, or moving between living with family and living alone, were not associated with an increased mortality risk, although there were some differences by gender. The institutional care sector in China is still in its infancy, with provision based on ability to pay market fees rather than need associated with age-related function impairment. The findings show that living in, or moving into, an institution is associated with a high mortality risk therefore requires further investigation in the context of a rapidly changing Chinese society.

## Highlights

• Examines the association between changes in living arrangements and survival status.
• Higher mortality risk for elderly people who stay in and move into an institution.
• Higher mortality for older men who move from an institution to the community.
• Higher mortality for older women who move to living alone.

## 1 Introduction

The living arrangements of older people are an important determinant of their health as well as their mortality (e.g. Feng, Jones & Wang, 2015; Gu, Dupre & Liu, 2007a; Lysack, Neufeld, Macneil & Lichtenberg, 2001; Zhang, 2015). In the context of rapid population ageing and decreasing family sizes, such arrangements are especially dynamic, particularly following changes in one's marital status (Freedman, 1996, Liang et al., 2005), socio-economic status (Martikainen, Nihtila & Moustgaard, 2008) or health status (mental or physical) (Kasper et al., 2010, Miller and Weissert, 2000, Wang et al., 2009). This is especially the case in China where the traditional family system of co-residence with adult children has come under pressure both as a result of rapid declines in fertility since the 1970 s (Zhao and Guo, 2010) and high levels of rural-urban migration throughout the last decade, resulting in an increasing number of older people living separately from their adult children (He and Ye, 2014). Living arrangements play a vital role in individuals’ capacity to provide support, and by extension they can also affect one's ability to meet their physical and social needs with the resources available to them, particularly as older people's physical or care needs often escalate, and their socioeconomic resources often decline, with age (Hays, 2002; Waite & Hugfies, 1999).

Previous research has revealed mortality differences depending on individuals’ living arrangements. For example, older people living with other household members have a lower mortality rate than those living alone due to receiving support with their daily care, as well as physical and emotional support (Lund, Due, Modvig, Holstein & Damsgaard, 2002). Conversely, living with other household members may encourage dependence and speed up the age-related loss of physical ability, while conflicts between older people and family/household members may increase the risk of poor health and mortality (Sereny and Gu, 2011, Zhou and Qian, 2008, Li et al., 2009). Older people living in institutions may receive professional personal care which may reduce the mortality risk, however such a living arrangement is associated with higher mortality rates than other living arrangements (Herm, Poulain & Anson, 2013), which may be due to the older person's poorer functional status (Gu et al., 2007a). The causal relationship between living arrangements transitions and mortality remains poorly understood, reflecting in part the lack of longitudinal data. At the same time, endogeneity is a challenge which is difficult to avoid when using cross-sectional data. Moreover, very few studies have compared the mortality risk between home and community residents on the one hand, with that faced by individuals living in institutions. The limited number of studies which have been conducted have been primarily in the USA and Europe, where institutional care is quite different compared to China and other emerging economies. To the best of our knowledge, there have been no such studies in China, despite China being home to the largest population of older people in the world.

More importantly, as social and family structures have changed rapidly, the living arrangements of older people are perceived as a dynamic process rather than a static status, which in turn may influence the adaptability to new circumstances, and thereby upon older people's mortality (Li and Li, 2015, Kasper et al., 2010). However, the association between changes in living arrangements and older people's mortality remains under-studied. This study aims to fill this gap, using unique longitudinal data stretching over a 10-year period in order to examine the effects of living arrangement transitions on the mortality of elderly persons in China in the first decade of the twenty-first century.

### 1.1 Living arrangements transition and mortality

The living arrangements of elderly people are subject to change, often in order to cater for their changing needs (Kasper et al., 2010). In certain cases, older people's living arrangements and their need for care are intertwined; for instance, when one's functional status deteriorates, an older person might move from living alone to living in an institution or joining their adult child's family (Korinek, Zimmer & Gu, 2011). In other instances, changes in living arrangements may be linked to one's own life events such as widowhood, or changes in the household composition (Korinek et al., 2011, Hays, 2002). Living alone has been shown to double one's odds of being admitted into an institution compared with living with one's spouse (Gaugler, Duval & Anderson, 2007).

Living arrangements, especially the change from a familiar environment to an unfamiliar one can have an impact on the risk of mortality in later life. For instance, Robards et al. (2014) found that a move into residential housing in the UK was associated with a higher risk of mortality within 1–2 years of the move, even after controlling for health status at the time of the move. The mortality risk also depends on the relationship between the carer and the older person, as older people who were cared for by a spouse, children or other relatives had a lower risk, compared to those with unrelated caregivers (Wang et al., 2009).

### 1.2 Living arrangements transition in China

The changing living arrangements among older people is an issue of increasing policy concern in China, where the world's largest ageing population resides. In 2013 there were 131 million people aged 65 and over, accounting for 9.7% of the total population ( National Bureau of Statistics (NBS), 2014). Living with family members remains the traditional living arrangement for older people so that they can receive care from their adult children or extended family (Gu et al., 2007a, Zimmer, 2005). However, due to rapid socioeconomic development, urbanization, and the one-child family policy, the structure of the family has been fundamentally altered recently (Wang, Cheng & Han, 2014), with implications for the availability of support towards older people. On the one hand, economic development may facilitate older individuals with a higher socioeconomic status to live independently, avoiding potential intergenerational conflict with family and enjoying a better quality of life compared to those living with children (Sereny and Gu, 2011, Zhou and Qian, 2008). On the other hand, such development may also enhance younger adults’ preference for independent living, leading to migration to urban areas or cities with higher economic development in order to find work and a better life, and resulting in the separation of older people from their adult children (Zeng and Wang, 2003, Phillips and Feng, 2015). Recent social and economic changes in China are reflected in the rapid increase in empty-nest elderly households; elders living alone or only with their spouse accounted for more than 38 percent of the total older population according to 5^th^ China's Census in 2000; however, in just a decade this had risen to nearly 50 percent or around 100 million Chinese elders (the 6th China's Census) (Sun, 2013).

Recent research shows that with increased age, individuals tend to make a transition into coresidence with children or within multigenerational households (Gu et al., 2009); at the same time older individuals find it difficult to care for themselves, and are more likely to co-reside with adult children (Ren & Treiman, 2015; Sereny, 2011). With rising life expectancy, more older people are surviving into their 80s and older; according to recent projections, the annual growth rate of the number of disabled elders will be more than one-third higher than that of the total elderly population between 2010–2050 (Zeng et al., 2015). On the other hand, living in an institution has increased slowly in China due to strong cultural norms encouraging familial care, and a limited provision of institutional care system (Gu et al., 2007a). In 2013, there were only 24.39 beds in elderly care institutions per 1000 senior citizens (NBS, 2014). Indeed, a key difference from western patterns is the provision of public institutional care for older people in rural China under the “Five Guarantees” scheme, and for older individuals in urban areas who face a “triple jeopardy” (also called the “three-no” category) of having no living family members; little or no income; and no physical ability to work. In such cases, the government has a responsibility for welfare provision in the form of food, clothing, fuel, education and burial expenses. As a result, public institutional care is targeted at the most disadvantaged older people, who face a triple jeopardy of poor health, inadequate income levels and weak social support networks, as well as a lower life expectancy (Phillips et al., 2010 p.218). In reality, the other side of the coin relates to healthy and young-old individuals living in urban well-facilitated nursing homes, where their needs are well catered for (Chu and Chi, 2008). Such older adults have a better health status rather than individuals with poor health who are in need. In addition, residential care in China is increasingly being extended to elderly parents of children who are unable to provide care but who can afford to purchase it. Elders in this category often wish to avoid causing trouble to their children and seek better institutional care than what could be provided at home, albeit at a high market price (Wong and Leung, 2012). Thus in the Chinese context, it is not clear-cut whether moving into (or out of) institutional care is associated with an elevated or reduced mortality risk.

### 1.3 Research question and theoretical framework

1.3

To-date, there are few studies on the impact of changing living arrangements among older people in China on their mortality risk, despite the clear policy implications of the issuer. This paper examines whether changes in living arrangements are associated with subsequent mortality risk for Chinese elders after controlling for other demographic, socio-economic and health status variables.

**Fig. 1:**
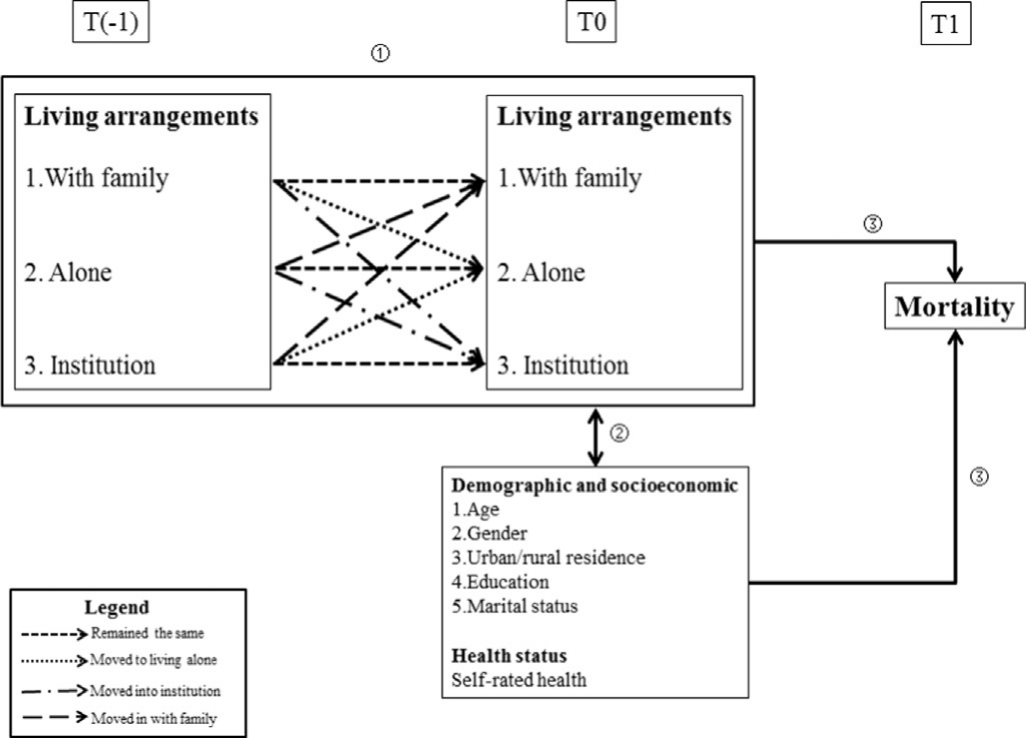
Theoretical framework of the links between changes in living arrangements and the risk of mortality.

Fig. 1 illustrates the paper's theoretical framework of the linkage between changes in living arrangements and the mortality risk. Changes in the living arrangements of older people are observed between T(-1) and T0. Three types of living arrangements are distinguished: living alone, living with family and living in an institution. Over the observation period, elderly people could remain in the same living arrangements (e.g. continuing to live with family members) or they could change their living arrangements (e.g. moving from living with family members to living alone) ①. Such changes could be either proactive or reactive, facilitating older people to meet the needs of their new situation (e.g. becoming widowed or remarrying; reporting better or worse health status) along with the process of becoming older at T0 ②. At the same time, changes in older people's living arrangements could force them to adapt to a new and unfamiliar environment, which could in turn influence their risk of mortality. The combination of changes in living arrangements, individual demographic and socio-economic characteristics, and health status at T0 can all influence the risk of mortality at T1 ③.

We hypothesise that older people who remain in the same living arrangements face a lower mortality risk as they are not exposed to a new environment and therefore need not adapt; by contrast, those who change their living arrangements may experience an adverse impact on their survival rate. For instance, moving from living alone to living in an institution may be associated with a higher mortality risk as a result of a deterioration of one's health and a disruption of one's social support networks. The aim of this paper is to consider such trajectories as well as to investigate the gender dimensions, drawing on existing literature which points to important differences in men's and women's living arrangements (Li et al., 2009, Gu et al., 2009) and in their survival status (Li et al., 2009, Gu et al., 2007a).

## 2 Data and methods

### 2.1 Data

This study uses the Chinese Longitudinal Healthy Longevity Survey (CLHLS), which collected extensive data on a large population of oldest-old individuals aged 80–112, with a comparative sample of younger elders aged 65–79. The survey is based on a randomly selected sample of elderly Chinese individuals from almost half of all the counties and cities of 22 out of 31 provinces in China. These areas cover 1.1 billion people, or approximately 85 percent of the total population in China. The original sample of 16,064 persons was interviewed in 2002 (T (-1) in Fig. 1) and 8,175 were re-interviewed in 2005 (T0) (2,015 respondents were lost to follow up in 2005). These 8,175 form our initial analytical sample, with their mortality observed in (T1). A total of 1,958 respondents were lost to follow-up after 2005, for whom we cannot determine whether they are alive or dead. In order to produce unbiased results, we assume that those lost to follow-up observations in terms of the survival status do not depend on the response outcome after taking account of the predictors, as would be expected in order to meet the assumption of Missing at Random (MAR) (Rubin, 1976). For those who were lost in post-2005 follow up waves, we found that age, gender, education, urban/rural residence, changes in living arrangements, and self-rated health in 2005 exhibited significant differences in predicting the loss to follow up from those whose survival status was known (either dead or alive). All these variables are included in the models which is required in order to satisfy the MAR assumption. Therefore, our final analytical sample is 6,191 respondents with sufficient data for the present analysis (excluding those lost to follow-up observations). The outcome variable of interest is death. A total of 3,703 respondents died between 2005 and the final observation point in 2011. (Appendix A shows the sample sub-categories with a status of survival, death, or lost to follow-up from the 2002, 2005, 2008 and 2011 waves). The 2002 wave included 4,984 respondents aged between 65–79 years old who were recruited in 1998 and 2000 and who replaced individuals from the oldest-old group aged 80 and over (Zeng, 2013).

### 2.2 Method

A Cox proportional-hazard regression model is applied to examine the association between changes in living arrangements between 2002 and 2005, and survival status through 2011 (total six years period). This model makes a parametric assumption concerning the effect of the predictors on the hazard function without making an assumption regarding the nature of the hazard function itself. It assumes that the predictors act collectively on the hazard function, but the hazard function is not assumed to remain constant in the model (Harrell, 2001).^1^ The model tests the assumption of proportional hazards by entering an iteration term consisting of the covariate times into a Cox regression model with the covariate (Harrell, 2001). All the models were estimated using the SPSS v.22 software.

*Mortality* is the outcome variable determining whether the respondent had died by the next wave. This information was collected from the respondent's next-of-kin and as such is subject to some measurement bias. For example, the mortality of those living alone may be underestimated as deceased respondents without kin could potentially be misclassified as lost to follow-up. It is not possible to correct for this, although it has been taken into account in interpreting the results.

*The changes in living arrangements* is an independent variable. There are three categories of living arrangements at the baseline: living with family members (not alone), living alone (alone), and living in an institution. These categories, firstly, allow us to understand the implications of living arrangements for older people's wellbeing and by extension their mortality risk (Davis et al., 1997); and secondly, they highlight the importance of living alone as a critical factor in terms of receiving social support. The changes in living arrangements between 2002 and 2005 were categorised as: unchanged not alone, unchanged alone, unchanged in institution, not alone to alone, not alone to institution, alone to not alone, alone to institution, institution to not alone, and institution to alone.

The other independent variables include individual demographic and socio-economic characteristics (age, gender, urban/rural residence, education and marital status), and one's health status. The education variable measures whether older people had completed formal education with “None” standing for not having received any formal education, and “educated” meaning that they had received at least one year of schooling. This distinction is appropriate for the particular cohorts under study, as it is estimated that 60% of people aged 60 and over are illiterate (Zhu and Xie, 2007). Marital status is recoded into four categories of married, separated or divorced, widowed, and single never married. Health status is measured through self-rated health (good, fair or poor, as well as a do-not-know category), which has been found to be a sensitive and reliable indicator of individuals’ current health status and mortality, particularly among elderly people (Wu and Schimmele, 2006). These variables are measured in 2005 in order to assess their independent effect on mortality after the change in living arrangements. This is especially important for health, where change may have triggered the living arrangements transition itself.

## 3 Results

### 3.1 Descriptive results

Table 1 presents descriptive information for the respondents’ survival status at T1 and predictor variables at T0 (2005) for the current sample. About 60% of the sample had died at T1 (2011). The majority of the analytical sample resided in rural areas (60.8%), had no formal schooling (59.6%) and were widowed (62.9%), while 43.7% reported good health. The living arrangements for more than four-fifths of the sample had remained unchanged from 2002 to 2005, and the majority of individuals lived with other people through the period (77.1%). In terms of gender differences, the percentages of deaths were similar (58.1% among men and 60.7% among women). The men in the sample tended to be younger than women, with about 67% of the male sample being in their 70 s or 80 s, and about 25% of men aged above 90 (52% and 40% of women respectively). These differences reflect gender differences in mortality (affecting the panel sample between 2002 and 2005), and gender differentials in life expectancy are also evident when comparing marital status as more than 50% of men were married (compared to less than 20% of women) and 78.7% of women were widowed (compared to 43.8% of men). The percentages of reporting good health were similar (43.7% of men and 45.9% of women). A higher proportion of women had received no formal schooling (82.1% compared to 32.6% of men). In terms of changes in living arrangements, men were more likely to continue living not alone (80.1% compared to 74.7% of women), while women were more likely to continue living alone (8.5% compared to 5.7% of men).

**Table 1:** Survival status from 2005 to 2011 and variables used in the analysis at T0 (2005).

	Both genders (n=6,191)	Male (n=2,815)	Female (n=3,376)
***Dependent variable***			
**Survival status (%)**			
Alive	40.5	41.9	39.3
Died	59.5	58.1	60.7
	
***Predictor Variables***			
		
*Demographic and socioeconomic*			
		
**Gender (%)**			
Male	45.5	–	–
Female	54.5	–	–
			
**Age (%)**			
60–69	6.9	7.4	6.4
70–79	30.4	35.0	26.6
80–89	29.4	32.7	26.7
90–99	20.7	18.8	22.4
100 and over	12.6	6.2	18.0
		
**Residence (%)**			
Urban	39.3	39.7	38.8
Rural	60.8	60.3	61.2
		
**Education (%)**			
No schooling	59.6	32.6	82.1
Some schooling	40.4	67.4	17.9
		
**Marital status**			
Married	33.5	50.3	19.5
Separated/ divorced	2.7	4.1	1.6
Widowed	62.9	43.8	78.7
Single never married	0.9	1.8	0.1
		
**Changes in living arrangements (%)**			
Unchanged not alone	77.1	80.1	74.6
Unchanged alone	7.2	5.7	8.5
Unchanged in institution	2.8	2.8	2.8
Not alone to Alone	6.0	5.6	6.3
Not alone to Institution	0.3	0.4	0.3
Alone to Not alone	5.9	4.8	6.8
Alone to Institution	0.3	0.4	0.2
Institution to not Alone	0.2	0.1	0.3
Institution to alone	0.1	0.1	0.2
	
**Self-rated health (%)**			
Good	43.7	45.9	41.8
Fair	31.1	32.4	30.1
Poor	17.7	16.6	18.6
Unable to Answer	7.5	5.1	9.5

In order to understand the changes in living arrangements among all individuals and between men and women, Table 2 presents the cross-tabulation of living arrangements in 2002 (row) and living arrangements in 2005 (column). About 92.4% of the respondents were not alone in 2002 and in 2005, with 7.2% changing from living with others to living alone, and 0.4% moving into an institution. Among those who lived alone in 2002, 44% moved to living with others, 53.8% continued to live alone and 2.2% moved into an institution. Interestingly, among those living in an institution, 7.7% moved to living with others, 3% moved to living alone and 89.3% continued living in an institution. A slightly lower percentage of men compared to women moved from living with others to living alone (6.5% and 7.7% respectively), whereas a higher percentage of men compared to women moved from living alone to living with others (44.2% and 43.9% respectively). Among individuals who were living in an institution in 2002, men were less likely than women to move in with others (4.7% of men compared to 9.9% of women), and men were also less likely than women to switch to living alone (1.2% of men compared to 4.5% of women). As a result, a higher proportion of men continued living in an institution compared to women (94.1% compared to 85.6%).

**Table 2:** Changes in living arrangements between 2002 and 2005.

			Living arrangement in 2005		
			Not Alone	Alone	Institution
Total	Living arrangement in 2002	Not Alone	92.4%	7.2%	0.4%
		Alone	44%	53.8%	2.2%
		Institution	7.7%	3.0%	89.3%
		Total	83.2%	13.3%	3.5%
					
Male	Living arrangement in 2002	Not Alone	93%	6.5%	0.5%
		Alone	44.2%	52.6%	3.2%
		Institution	4.7%	1.2%	94.1%
		Total	85%	11.4%	3.6%
					
Female	Living arrangement in 2002	Not Alone	92%	7.7%	0.3%
		Alone	43.9%	54.6%	1.5%
		Institution	9.9%	4.5%	85.6%
		Total	81.8%	14.9%	3.3%

**Fig. 2:**
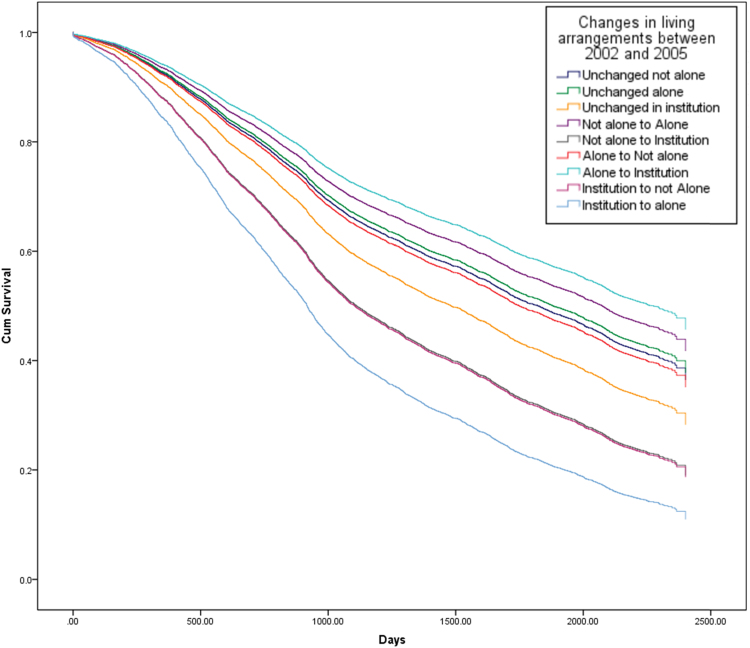
Survival curves from 2005 to 2011, by changes in living arrangements between 2002 and 2005 (whole sample) (Note: the lines for elderly people who moved from “not alone to institution” and from “institution to not alone” overlap in this figure and are shown by the second line from the bottom).

**Fig. 3:**
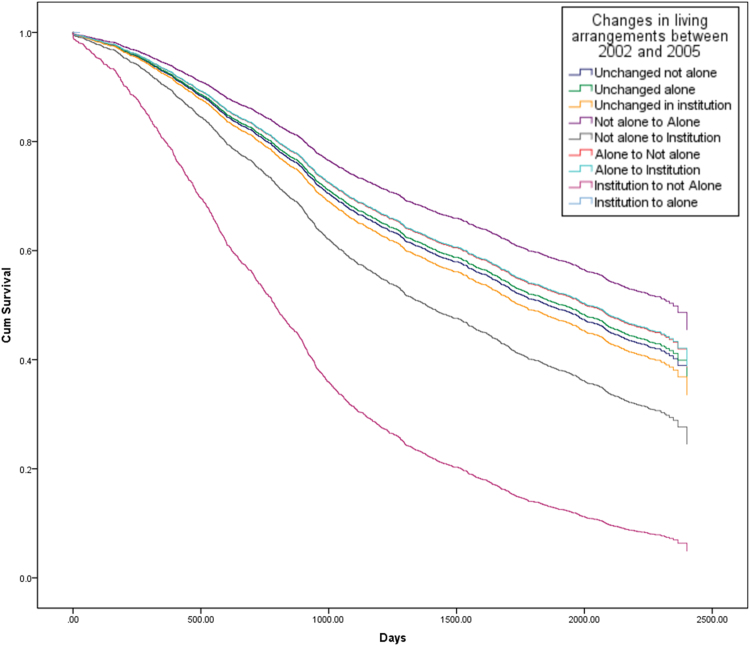
Survival curves from 2005 to 2011, by changes in living arrangements between 2002 and 2005 (male) (Note: the line for elderly people who moved from an “institution to alone” is omitted in this figure due to low cell counts for this category in the model).

**Fig. 4:**
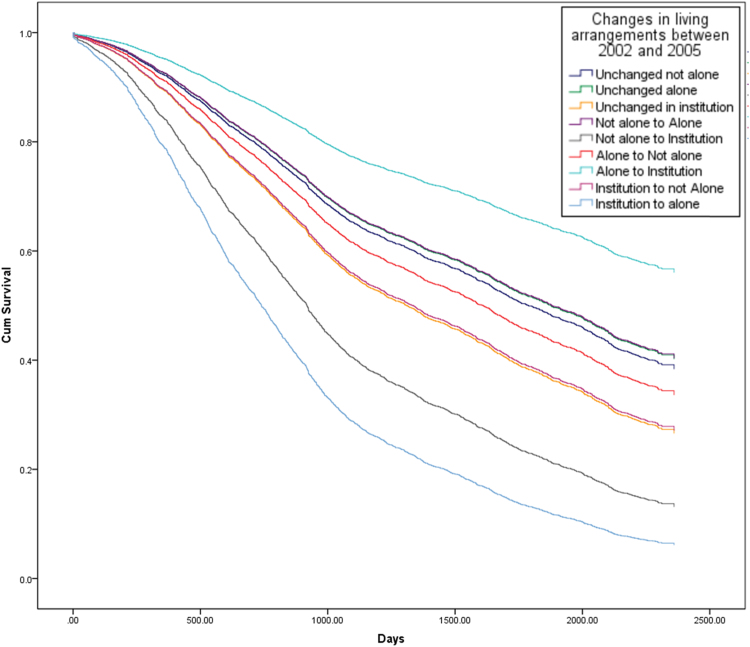
Survival curves from 2005 to 2011, by changes in living arrangements between 2002 and 2005 (female) (Note: the lines for elderly people who were “unchanged not alone”, “Unchanged alone” and who moved from “not alone to alone” overlap in this figure).

Fig. 2, Fig. 3, Fig. 4 show the survival curves associated with different trajectories of changes in living arrangements for both genders (Fig. 2), followed by males and females separately (Fig. 3, Fig. 4). The figures show the cumulative proportion surviving at a particular time, which is measured in days. From Fig. 2, it is clear that older people who continued living in an institution, who moved from an institution to living with others, or who moved from an institution to living alone showed a lower cumulative proportion surviving than those experiencing other changes in their living arrangements. Less than 30% of older people who continued living in an institution or who moved from an institution to living not alone, were still alive at T1, as were about 10% of those who had moved from an institution to living alone during this time.

Focusing on men, it can be seen that the survival curve for those who had moved from an institution to living not alone is significantly lower than for those who had experienced other changes in their living arrangements, and less than 5% of older men were alive in 2011 having undergone such a change in their living arrangement between 2002 and 2005 (Fig. 3). By contrast, the survival curves for women show a more diverse picture, with older women who had moved from living alone to moving into an institution showing the highest cumulative proportion surviving compared to women experiencing any other change in their living arrangements, as about 60% of this group were still alive at T1. In contrast, elderly women who had moved from an institution to living alone had the lowest survival rate at T1 (about 5%) (Fig. 4).

### 3.2 Cox-regression results

The Cox-regression results generally confirm the findings of Fig. 1, Fig. 2, Fig. 3 and are shown in Table 3. After adjusting for individual demographic and socio-economic characteristics, and health status, the analysis found that continuing to live in an institution and moving into an institution from living with others were linked to a greater risk of dying than continuing to live with others, for both men and women (Hazard Ratios are 1.25 and 1.65 respectively). For older men, moving to live with family from an institution was associated with a higher risk of dying than continuing to live with family (HR=2.92), while changing from living with family to living alone was associated with a lower risk of dying than continuing to live with family (HR=0.76). Among older women, moving from an institution to living alone, or continuing to live in an institution, or moving from living with family to an institution, were all associated with a greater risk of dying than continuing to live with family (HR= 2.92, 1.38 and 2.12 respectively).

**Table 3:** Results of Cox-proportional model analysis of mortality risk.

	Both genders		Male		Female	
	HR	95% CI	HR	95% CI	HR	95% CI
**Age**						
60–69 (ref:)						
70–79	2.07***	(1.62–2.65)	2.47***	(1.73–3.51)	1.73**	(1.22–2.45)
80–89	4.99***	(3.92–6.36)	5.96***	(4.20–8.44)	4.17***	(2.97–5.88)
90–99	9.46***	(7.40–12.11)	9.69***	(6.78–13.85)	9.06***	(6.42–12.78)
100 and over	15.09***	(11.71–19.43)	16.55***	(11.32–24.18)	13.86***	(9.76–19.67)
**Female (ref: Male)**	0.78***	(0.72–0.84)				
**Educated (ref: Non-Educated)**	1.01	(0.93–1.10)	1.01	(0.91–1.12)	1.02	(0.90–1.15)
**Rural (ref: Urban)**	0.99	(0.93–1.06)	0.99	(0.89–0.99)	0.99	(0.90–1.08)
					
**Marital status**						
					
Married (ref)						
Separated/ divorced	1.11	(0.89–1.38)	1.32*	(1.03–1.70)	0.67	(0.42–1.08)
Widowed	1.26***	(1.15–1.38)	1.34***	(1.19–1.51)	1.18*	(1.00–1.38)
Single never married	1.67**	(1.19–2.34)	1.75**	(1.19–2.57)	2.18	(0.88–5.35)
				
**Living arrangements**						
				
Unchanged not alone (ref:)						
Unchanged alone	0.96	(0.85–1.10)	0.97	(0.79–1.21)	0.95	(0.81–1.12)
Unchanged in institution	1.25*	(1.05–1.50)	1.06	(0.79–1.42)	1.38**	(1.10–1.74)
Not alone to Alone	0.87	(0.75–1.00)	0.76*	(0.61–0.95)	0.95	(0.78–1.15)
Not alone to Institution	1.65*	(1.04–2.63)	1.36	(0.73–2.55)	2.12*	(1.05–4.26)
Alone to Not alone	1.04	(0.91–1.18)	0.92	(0.74–1.14)	1.14	(0.96–1.35)
Alone to Institution	0.78	(0.43–1.41)	0.92	(0.43–1.95)	0.60	(0.23–1.62)
Institution to not Alone	1.66	(0.96–2.87)	2.92*	(1.08–7.90)	1.36	(0.71–2.63)
Institution to alone	2.19	(0.91–5.28)	–	–	2.92*	(1.21–7.05)
					
**Self-rated health**						
					
Good (ref:)						
Fair	1.18***	(1.09–1.27)	1.26***	(1.12–1.41)	1.10	(0.99–1.23)
Poor	1.79***	(1.64–1.96)	2.07***	(1.81–2.36)	1.62***	(1.44–1.83)
Don’t know	1.87***	(1.67–2.09)	2.21***	(1.82–2.69)	1.67***	(1.45–1.93)

“--” stands for odd values due to low cell counts for this category.

*** p<0.001

** p<0.01

* p<0.05

Age and health status at T0 were important for the survival status of elderly people at T1. The older the individuals, the higher their mortality risk. Centenarians were more than 15 times more likely to die than elderly people aged in their 60 s. Women showed a lower risk of mortality than men (HR=0.78), while widowed or never married elders had a higher risk of mortality than those who were married (HR =1.26 and 1.57 respectively). As might be expected, the results indicate that those in poorer health show a higher risk of dying. Elderly people with fair health or poor health were 1.18 and 1.78 times more likely to die than those with good health respectively. In the separate models by males and females, the results are broadly similar, namely the older one's age and the worse one's health, the higher their mortality risk. Widowed men and women were at a higher risk of dying than those who were married, with the risk of dying among never married men being 1.75 times the risk among married men. There are no significant effects of one's education level and their urban/rural residence on their risk of mortality.

## 4 Discussion

This study has investigated the association between changes in living arrangements and survival among elderly people in China. The results show that changes in living arrangements are associated with subsequent risk of mortality, and that there are significant gender differences permeating this relationship. The results support the initial hypothesis stated. For both genders, there is no significantly different effect on the mortality risk of continuing to live in the community (either with family members or alone) or changing one's living arrangements within the community (e.g. shifting between living with family and living alone). By contrast, continuing to live in an institution or transitioning from living with family members to living in an institution are both associated with a higher mortality risk than continuing to live with one's family. In addition, for elderly men, moving out of an institution to live with family members, and for women, moving out of an institution to live alone, are also linked with a higher mortality risk.

Few studies so far have indicated differences in the mortality of older people according to their changing living arrangements between institutions and their home, highlighting that changes in living arrangements raise the risk of mortality (Li and Li, 2015). In particular, previous literature has shown that changing from living with family members to either living alone or living with one's spouse only results in a higher mortality risk for older people (Davis et al., 1997, Koskinen et al., 2007). The original contribution of this study relates to providing a holistic picture of the effect of changes between different types of living arrangements on mortality among Chinese older people, focusing especially on the transition between a nursing home and their own home. The following discussion might explain the reasons of these key findings.

### 4.1 Higher mortality risk for elderly people who stay in and move into an institution

The advantages of living in an institution, such as better institutional care and the alleviated effect on strains and pressures that may be caused by living together with family, may present “buffering” effects for the health outcomes of elderly people (Zhou and Qian, 2008). Indeed, previous studies in the Chinese context using cross-sectional data reported that living in institutions was associated with a lower mortality risk than living alone among oldest-old Chinese people (Sun and Liu, 2006), and that institutional living lowers the mortality risk for men compared with living alone, living with children or with others (Li et al., 2009). However, continuing to live in an institution is associated with a higher risk of dying in the present longitudinal study, which may indicate inadequate provision towards elderly people in institutional settings, as such provision in China is still based on the principle of destitution or elderly people's ability to pay market fees for services rather than on the need arising from age-related functional impairment (Wong and Leung, 2012). Older people who moved from living with family to an institution show a higher mortality risk than those continuing to live with family. This finding is consistent with a study in the UK indicating a higher risk of mortality among elderly people who moved to residential housing from their private homes (Robards et al., 2014). Such change may be due to the loss of families’ ability to care for elderly relatives, which may be linked to the deterioration of the older person's health (Gu et al., 2007a). Indeed, adjusting to a new environment has been shown to be more difficult for elderly compared to younger adults (Robert and Li, 2001), and this could be contributing to an increase in the mortality risk among elderly people. In contrast, there are signs of the “protective” impact of changing living arrangements on the mortality risk of elderly women, as those who moved from living alone into an institution faced a lower risk than those continuing living with family (although such results are not statistically significant).

Elders who continued living in institutions and those who moved from living with family into institutions tend to report poorer health compared with other groups (Appendix B). Over 28 percent of elders who continued living in institutions reported difficulty with at least one ADL, compared with almost 43 percent of those who shifted from living with family into institutions. These proportions compare with just over 24 percent and 11 percent of elders living with family or alone respectively. In addition, the proportion of persons living in or moving into institutions who report difficulty with more than three ADLs or IADLs is even larger, which might be a key contributing factor to their mortality risk.

Uniquely for the Chinese context, the erosion of the family care system in modern society has been accompanied by the rapid development of institutional care as a result of the equally rapid growth of long-term care needs. However, the quality of care provided in institutions is relatively low compared to western societies, as such care organisation is still at an embryonic stage, and is restricted by limited economic and labour resources (Wu et al., 2008). At the end of 2014, each thousand elders aged 60 and over corresponded to 27.2 institutional beds, and only 1.36 percent of the total older population lived in institutions specialising in care for older people (Ministry of Civil Affairs, 2015). Such patterns reflect a limited capacity for the delivery of effective long-term care services, which may in turn result in frail elderly persons in institutions not currently benefitting from the system of formal care provision, and being exposed to a higher mortality risk.

### 4.2 Gender differences: higher mortality for older men who move from an institution to the community and for older women who move to living alone

Although the results on the impact of changes in living arrangements on the mortality risk are broadly similar for the separately models of older men and women, it should be noted that elderly men who moved from an institution to living with family, and elderly women who moved from an institution to living alone, show significantly higher mortality risks. The former risk among men may reflect the Chinese culture regarding preferences for one's place of death, as death at home brings physical and emotional comfort, a sense of belonging and safety, and an increase in autonomy and privacy (Tang, 2000). Given the sense of “a wandering soul with no place to rest” of death outside the home, most elderly Chinese people prefer to reach the end of their life at home (excluding those who do not have offspring) (Gu et al., 2007b). Therefore, elderly men who moved from an institution to living with family may expect to die within a short period, raising the mortality risk in this category (50% of this category died at home at T1 (This information comes from exploring the CLHLS dataset)). An alternative explanation may relate to the study by Gu et al. (2007a) who found that age is negatively associated with institutionalization among oldest-old Chinese persons. In this study, the higher mortality risk among elderly men who moved from an institution to living with family can be interpreted as elderly men wanting to move out from the institution as they become older. However, the lower level of professional care service provided by family members, and intergenerational conflict with family member could increase the risk of mortality while living with family, and reduce such risk when moving to living alone, a finding which is supported by previous research (Davis et al. 1997).

Elderly women's move from an institution to living alone could result in a high degree of vulnerability in later life, illustrated by a higher mortality risk. There were no childless women in the sample; nevertheless, we lack information on the reasons behind their moving out of an institution but not with their offspring's family. One possible reason could be the unaffordable market fees for the services provided in an institution that drives women to move out. Prior research has also indicated a strong association between income and institutionalization (Martikainen et al., 2008). Comparison of the household income between elders who move into and out of institutions confirms that the latter group is much more vulnerable with an average income of Chinese Yuan (CNY) 12,954 per year compared to CNY 18,848 per year among those who moved from an institution to living alone. Older individuals who continued living in an institution had an average income of CNY 24,171, and those who moved into institutions from living with family reported the highest annual income of CNY 30,645. Such patterns may be contextualised in recent policy developments, including decentralization and marketization which have resulted in a reduction of the government's investment in aged-care institutions, forcing such institutions in turn to become financially self-reliant. Although some institutions may still receive partial funding from the government, most have to source their own resources in order to balance their costs (Zhan et al., 2006), for instance from elders and their families. Such developments can force elders with low incomes, excluding those belonging to the “Five Guarantees” or “triple-jeopardy” categories, to move out of institutions due to the lack of funds.

At the same time, as many long-term care institutions in China are ill-equipped to provide high-demanding care to older people, severely disabled individuals are often forced to move back into their home, with “for profit” institutions then being in a position to admit healthier residents. In terms of admissions, public institutions have three common exclusion criteria that include infectious diseases, mental illness (including dementia), and functional dependency (e.g. being partly or totally bed-ridden) (Wu et al., 2008). Indeed, avoiding high mortality is critical for such institutions, as legislation and regulations on long-term care provision is still immature in China, and potential conflicts with the families of older residents could result in the loss of future demand.

### 4.3 Limitations

Five potential limitations have been identified in this paper. The sample in this study was based on elderly people who were alive at T0 (2005) and whose survival status was measured at T1 (2008/2011). However, the dataset lacks information exploring the association between changes in living arrangements and mortality for elderly people who died before the survey was conducted at T0. The second limitation is we may be underestimating the actual transition probability/rate due to the measure of living arrangements being based on the survey data collection points. Unfortunately, the dataset does not provide information about changes in living arrangements in the intervening periods between the data points. A third limitation is the lack of information about the timing of the transition in one's living arrangements; indeed, the variation in the duration of one's new living arrangements could contribute to different risks of mortality (i.e. Robards *et al*., 2014). The fourth limitation relates to the respondents who are lost to follow up, who amount to 23 percent of the sample post-2005. It was not possible to impute survivorship for these individuals as although we might impute whether they were alive or dead, then imputing survival time was beyond this paper. One option considered would be to treat their survival time as the observation window; however, this approach was rejected as it involves a strong assumption that individuals in this group are alive in the next wave. This limitation has been mitigated here as all predictor variables were included in order to help satisfy the MAR assumption. The fifth and final limitation relates to the relatively rare occurrence of transitions from institutions to living alone for male respondents, which barred us from being able to explore such effect due to small sample sizes. Despite these limitations, we remain convinced that this study provides important new contributions to our understanding of the impact of changing living arrangements on the mortality risk of elderly people in China.

## 5 Conclusions and policy implications

In general, elderly persons who are older, male and in poorer health face a higher mortality risk than younger, female persons in better health. Consistent with other studies, one's education level and urban/rural residence have no significant effects on the risk of mortality (Feng et al., 2015, Li et al., 2009, Sun and Liu, 2006). The protective effect of marriage on the risk of mortality is consistent with existing results from both the West (Manzoli et al., 2007) and China (Feng et al., 2015, Li et al., 2009). We also considered the effect of income on the mortality risk, and the interactions between changes in living arrangements on the one hand, and demographic, socio-economic status and health status on the other hand; however, no significant differences were found.

In summary, older people who continue to live with family, or to live alone, and those who interchanged between living with family and living alone, do not show a significantly elevated mortality risk. However, continuing to live in, or moving into, an institution was associated with an increased mortality risk, as was moving out of an institution to live with family or alone.

In the coming decades, China will face rapid population ageing, resulting in a challenge to the family's ability to support older persons (Phillips and Feng, 2015). Developing institutionalized care could help relieve families’ responsibility for long-term care to their older parents. With the growth of the older population, the percentage of older Chinese persons living in institutions will undoubtedly rise in the 21^st^ century (Leung, 2010); however, the growth of beds in elderly care institutions at present has been much lower than the growth of the older population (NBS, 2014). This suggests that the current system needs to expand in order to meet the growing demands of older people who do not belong to the “triple-jeopardy” category, but who still require care. The quality of institutions also requires improvement in order to reduce the risk gaps between continuing to live in an institution and other living arrangements. Better care quality in institutions could also help to reduce the mortality risk for older people who moved from living with their family into an institution, helping them to adapt to their new environment, but such quality improvement requires efforts from national and local governments, and institutions.

As long as there is a higher preference of “ageing in place”, the majority of older people in China will remain living in the community rather than institutions. Policy interventions could further enhance community and home-based long-term care services in order to improve individuals’ well-being in later life. An example of such practice exists in Zhejiang province, which is one of the richest regions in the country, where a subsidy plan for long-term care was recently launched alongside a comprehensive evaluation system (Dong, 2012). As part of this system, both institutional and community care for disabled elderly people is being organised in order to offer allowances to individuals according to their degree of disability, their household income, and living arrangements. However, such initiatives are still at an early stage and need to be expanded to the whole country. In addition, the establishment of professional hospice care in order to provide high quality end-of-life care, is also emerging as a policy priority, especially for those individuals facing a ‘triple jeopardy’. More empirical research is urgently needed to inform the design of culturally appropriate services for older people in China.

## Figures and Tables

**Fig. 5:**
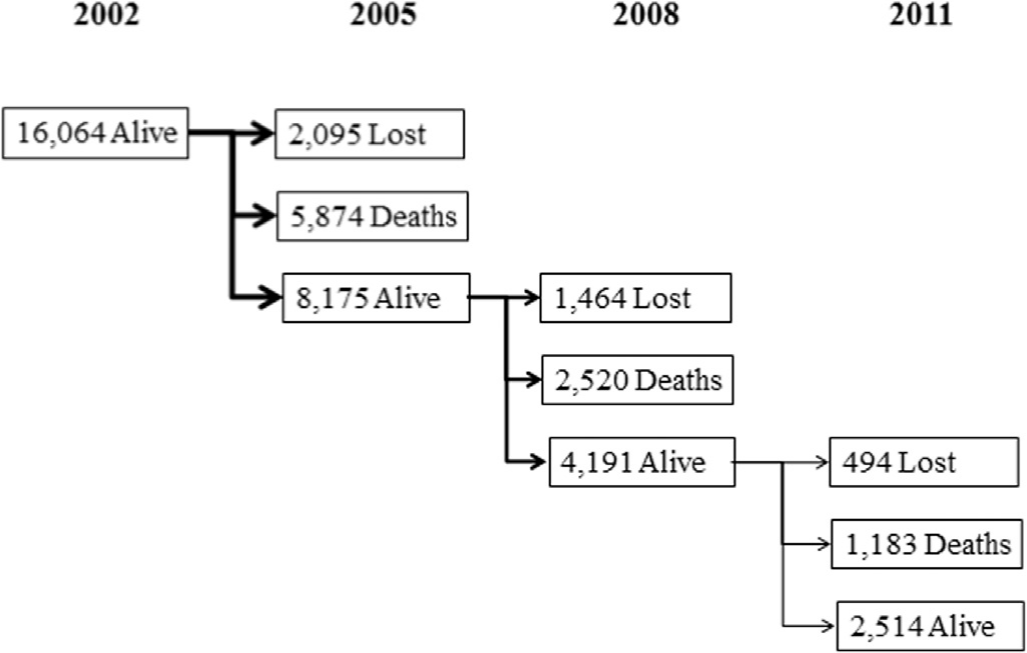
Status of survival, death, or lost to follow-up from 2002, 2005, 2008 and 2011 waves. Source: CLHLS longitudinal dataset from 2002 to 2011/2012. Total sample is 6,191 with sufficient data for the present analysis (21 missing in education, 2 missing in living arrangements, and 3 missing in the actual date of dead).

**Table B1:** Characteristics of changes in living arrangements for elderly people in 2005.

	Age	Gender		Residence		Education		Income
	Mean (range)	Male	Female	Urban	Rural	Non-educated	Educated	mean
Unchanged not alone	84.6 (65–120)	47.1	52.9	39.4	60.6	58.2	41.8	10873.9
Unchanged alone	84.3 (67–108)	36.2	63.8	32.6	67.4	70.1	29.9	13215.1
Unchanged in institution	88.5 (66–107)	45.2	54.8	65.5	34.5	64.4	35.6	24170.9
Not alone to Alone	83.8 (67–113)	42.7	57.3	36.3	63.7	59.4	40.6	14992.4
Not alone to Institution	89.5 (67–106)	57.1	42.9	66.7	33.3	52.4	47.6	30645.2
Alone to Not alone	87.2 (67–100)	37	63	34.2	65.8	65.8	34.2	14140.9
Alone to Institution	84.6 (68–104)	55.6	44.4	44.4	55.6	55.6	44.4	26445.8
Institution to not Alone	91.9 (81–106)	26.7	73.3	66.7	33.3	73.3	26.7	12953.9
Institution to alone	85.5 (78–100)	16.7	83.3	50	50	66.7	33.3	18848.0

(Continued) appendix B											
	Marital status				Self-rated health				ADLs		
	Married	Separated	Widowed	Never married	Good	Fair	Poor	DN	Without any assistance	Needs 3 assistance	More than 3 assistance
Unchanged not alone	42.2	2.3	55.3	0.2	44.2	31.4	16.6	7.8	75.8	14.9	9.3
Unchanged alone	–	3.3	93.3	3.3	41.3	28.6	25.0	5.1	88.6	7.4	4.0
Unchanged in institution	3.4	6.8	76.8	13	42.9	30.5	17.5	9.0	71.6	15.9	12.5
Not alone to Alone	1.9	4.8	92.5	0.8	40.6	33.6	22.6	3.2	84.9	8.1	7.0
Not alone to Institution	14.3	4.8	81	–	66.7	9.5	14.3	9.5	57.1	14.3	28.6
Alone to Not alone	11.1	2.7	85.6	0.5	43.8	28.8	18.5	9.0	70.4	17.1	12.5
Alone to Institution	–	–	77.8	22.2	50.0	16.7	27.8	5.6	88.9	5.6	5.6
Institution to not Alone	13.3	6.7	80	–	13.3	26.7	40.0	20.0	46.7	20.0	33.3
Institution to alone	–	–	100	–	33.3	66.7	0.0	0.0	100.0	0.0	0.0

## Appendix A

See Fig. 5 here.

## Appendix B

See Table B1 here.

## Footnotes

1 Parametric models were also tested and the results are presented in the supplementary analysis for comparative purposes (see supplementary material). The underlying pattern of results was unchanged.
