# Supplementary material for: Changes in living arrangements and mortality among older people in China
Source: SSM Popul Health. 2016 Nov 30;3:9–19. doi: 10.1016/j.ssmph.2016.11.009 (PMC5768996; doi:10.1016/j.ssmph.2016.11.009)
Supplement: Supplementary file 1 — Supplementary material [file mmc1.docx]

**(Online material) Results of logistic regression model analysis of mortality**

|  | Both genders | | Male | | Female | |
| --- | --- | --- | --- | --- | --- | --- |
|  | ORs | 95% CI | ORs | 95% CI | ORs | 95% CI |
| **Age** |  |  |  |  |  |  |
| 60-69 (ref:) | |  |  |  |  |  |
| 70-79 | 2.26*** | (1.71-2.99) | 2.88*** | (1.93-4.31) | 1.8** | (1.22-2.65) |
| 80-89 | 7.44*** | (5.60-9.89) | 9.93*** | (6.59-14.98) | 5.66*** | (3.80-8.44) |
| 90-99 | 27.06*** | (19.66-37.24) | 27.47*** | (17.16-43.97) | 24.96*** | (16.11-38.67) |
| 100 and over | 88.14*** | (56.64-137.17) | 115.79*** | (46.55-288.02) | 72.82*** | (42.35-125.22) |
| **Female (ref: Male)** | 0.63*** | (0.54-0.73) | |  |  |  |
| **Educated (ref: Non-Educated)** | 0.93 | (0.80-1.07) | 0.9 | (0.74-1.09) | 0.96 | (0.76-1.19) |
| **Rural (ref: Urban)** | 1.05 | (0.92-1.19) | 1.06 | (0.88-1.27) | 1.02 | (0.85-1.22) |
| **Marital status** | |  |  |  |  |  |
| Married (ref) | |  |  |  |  |  |
| Separated/ divorced | 1.38 | (0.96-1.98) | 1.86** | (1.19-2.91) | 0.74 | (0.37-1.48) |
| Widowed | 1.38*** | (1.18-1.62) | 1.62*** | (1.30-2.01) | 1.19 | (0.94-1.50) |
| Single never married | 2.47** | (1.24-4.91) | 2.92** | (1.38-6.18) | -- | -- |
| **Changes in Living arrangements** | | |  |  |  |  |
| Unchanged not alone (ref: ) | | |  |  |  |  |
| Unchanged alone | 0.96 | (0.76-1.23) | 0.86 | (0.57-1.29) | 1.01 | (0.75-1.36) |
| Unchanged in institution | 1.53* | (1.01-2.32) | 1.01 | (0.56-1.83) | 2.23* | (1.19-4.18) |
| Not alone to Alone | 0.73* | (0.56-0.95) | 0.66* | (0.44-0.98) | 0.77 | (0.54-1.08) |
| Not alone to Institution | 3.59 | (0.86-15.03) | 2.26 | (0.43-11.93) | 6.53 | (0.56-76.21) |
| Alone to Not alone | 0.99 | (0.76-1.30) | 0.92 | (0.59-1.42) | 1.04 | (0.74-1.46) |
| Alone to Institution | 0.84 | (0.27-2.64) | 0.92 | (0.20-4.19) | 0.65 | (0.11-3.88) |
| Institution to not Alone | 1.64 | (0.35-7.75) | -- | -- | 1.12 | (0.21-5.89) |
| Institution to alone | 3.61 | (0.40-32.66) | -- | -- | -- | -- |
| **Self-rated health** | |  |  |  |  |  |
| Good (ref: ) | |  |  |  |  |  |
| Fair | 1.27*** | (1.10-1.46) | 1.4*** | (1.15-1.70) | 1.15 | (0.95-1.41) |
| Poor | 2.66*** | (2.23-3.16) | 3.23*** | (2.48-4.21) | 2.27*** | (1.80-2.86) |
| Don’t know | 4.14*** | (2.85-6.02) | 5.18*** | (2.69-9.97) | 3.53*** | (2.23-5.58) |

***p<0.001; **p<0.01; *p<0.05

“--” stands for odd values due to low cell counts for this category.

Source: CLHLS, changes of living arrangements between 2002 and 2005, survival status in 2008 and 2011/2012. Total sample is 6,191.
